# Locked down by inequality: Older people and the COVID-19 pandemic

**DOI:** 10.1177/00420980211041018

**Published:** 2021-09-06

**Authors:** Tine Buffel, Sophie Yarker, Chris Phillipson, Luciana Lang, Camilla Lewis, Patty Doran, Mhorag Goff

**Affiliations:** The University of Manchester, UK; The University of Manchester, UK; The University of Manchester, UK; The University of Manchester, UK; Newcastle University, UK; The University of Manchester, UK; The University of Manchester, UK

**Keywords:** age-friendly cities, ageing populations, austerity, COVID-19, deprived neighbourhoods, older people, social exclusion, social inequality

## Abstract

This paper develops the argument that post-COVID-19 recovery strategies need to focus on building back *fairer* cities and communities, and that this requires a strong embedding of ‘*age-friendly*’ principles to support marginalised groups of older people, especially those living in deprived urban neighbourhoods, trapped in poor quality housing. It shows that older people living in such areas are likely to experience a ‘double lockdown’ as a result of restrictions imposed by social distancing combined with the intensification of social and spatial inequalities. This argument is presented as follows: first, the paper examines the disproportionate impact of COVID-19 on *older people*, highlighting how the pandemic is both creating new and reinforcing existing inequalities in ageing along the lines of gender, class, ethnicity, race, ability and sexuality. Second, the paper explores the role of *spatial inequalities* in the context of COVID-19, highlighting how the pandemic is having a disproportionate impact on deprived urban areas already affected by cuts to public services, the loss of social infrastructure and pressures on the voluntary sector. Finally, the paper examines how *interrelated social inequalities at both the individual and spatial level* are affecting the lives of older people living in deprived urban neighbourhoods during the pandemic. The paper concludes by developing six principles for ‘age-friendly’ community recovery planning aimed at maintaining and improving the quality of life and wellbeing of older residents in the post-pandemic city.

## 摘要

本文提出了这样一种观点，即后疫情时代的经济复苏战略需要侧重于建设更公平的城市和社区，而这又需要大力贯彻“关爱老年人”的原则，支持边缘化的老年人群体，特别是那些生活在贫困的城市社区、受困于劣质住房的老年人。我们表明，由于保持人际距离的要求以及社会和空间不平等的加剧，生活在这些地区的老年人可能会经历“双重封锁”。我们的论证如下：首先，本文探讨了新冠肺炎对老年人不成比例的影响，强调疫情如何在性别、阶级、族裔、种族、能力和性方面造成新的老龄化不平等、并加剧现有的老龄化不平等。其次，本文探讨空间不平等在新冠肺炎背景下的影响，强调疫情如何对贫困城市地区产生不成比例的影响，这些地区原本就已经受到了公共服务削减、社会基础设施损失和对志愿者服务部门的压力的影响。最后，本文探讨在疫情期间，个人和空间层面的社会不平等（两者相互交织）如何影响生活在贫困城市社区的老年人的生活。本文最后提出了“关爱老年人”的社区恢复规划的六项原则，这些原则旨在维持和改善后疫情时代城市老年居民的生活质量和福祉。

## Introduction

This paper makes the argument for developing ‘age-friendly’ recovery strategies in the context of growing inequalities affecting urban neighbourhoods. It does this by exploring the social impact of the COVID-19 pandemic, with a particular focus on issues facing older people living in urban environments. This goal should be seen in the context of population ageing and urbanisation, both identified as among the most significant social trends of the 21st century. Population ageing is taking place across all countries of the world, raising major issues for the direction of public policy. By 2050, one in six people will be 65 and over (16%), a steep increase from 1 in 11 in 2019 (9%). In Europe and Northern America, one in four people are expected to be aged 65 or over by 2050 (United Nations [UN], 2019). Of equal importance has been the continuing spread of urbanisation, with 55% of the world’s population now living in urban environments (UN, 2019). The relationship between these two major trends – ageing and urbanisation – is now the subject of increased academic and policy analysis. Urban environments create many advantages for older people, for example through providing access to cultural activities, leisure facilities and specialist medical care (Phillipson and Buffel, 2020). At the same time, they may also produce feelings of insecurity, arising from the impact of urban regeneration, population turnover and climate change (Lewis and Buffel, 2020; Wallace-Wells, 2019).

The pressures associated with urban living indicate challenges for policies seeking to reconcile population ageing with urban development (Buffel and Phillipson, 2016). Policies in Europe have emphasised the role of the local environment in promoting ‘ageing in place’, a term used to describe the goal of helping people to remain in their own homes and communities (rather than residential care) in later life (Wiles et al., 2012). The World Health Organization (WHO, 2007) has been especially influential in raising awareness about how to adapt urban environments to the needs and preferences of people ageing in place, through the development of its ‘Age-Friendly Cities’ project. Alley et al. (2007: 4) define an age-friendly city as a ‘place where older people are actively involved, valued, and supported with infrastructure and services that effectively accommodate their needs’. In 2010, the WHO launched the Global Network of Age-Friendly Cities and Communities (GNAFCC), which by the end of 2020 had reached a membership of around 1114 cities and communities in 44 countries (Rémillard-Boilard et al., 2021). The period from the mid-2000s saw a substantial growth of interest in age-friendly issues, with a variety of projects and achievements linking ageing populations to the need for changes to the built and social environment, transportation, housing and neighbourhood design (Moulaert and Garon, 2016; Stafford, 2019; van Hoof et al., 2021; WHO, 2018). However, a combination of widening inequalities within and between urban environments, and the impact of austerity on local government and city budgets, has raised questions about future progress in age-friendly and related activities (Buffel et al., 2018).

To these pressures may now be added the impact of COVID-19, with the pandemic having its greatest impact on areas characterised by high levels of deprivation, often with ageing populations, poor quality housing and communities experiencing long-term decline through de-industrialisation (Beatty and Fothergill, 2021; Marmot et al., 2021; Phillipson et al., 2021; Sun et al., 2021). This discussion focuses on the different types of disadvantage faced by older adults who are ageing in place in urban environments during the pandemic. The paper argues that under social distancing guidelines, older people living in socio-economically deprived urban neighbourhoods experience a ‘double lockdown’ as a result of interrelated social and spatial inequalities associated with COVID-19. This argument will be developed as follows: first, the paper examines the disproportionate impact that the pandemic is having on the lives of *older individuals*, further deepening inequalities along the lines of gender, class, ethnicity, race, ability and sexuality. Second, the paper examines the role of *neighbourhood inequalities* in the context of COVID-19, highlighting how the pandemic is having a disproportionate impact on deprived urban areas already affected by cuts to public services and the loss of social infrastructure (Marmot et al., 2020; Yarker, 2019). Finally, the paper explores how *interrelated social inequalities at both the individual and spatial level* are affecting the lives of older people living in deprived urban neighbourhoods during the pandemic, and develops six principles for ‘age-friendly’ community recovery planning in cities. By bringing together perspectives from urban studies with research on ageing from social gerontology, this paper offers a novel analysis of the ways in which the COVID-19 pandemic is creating new and reinforcing existing inequalities in the ageing population.

## The impact of COVID-19 on older people

Older people have been disproportionately affected by COVID-19, whether in hospital, the community or in care homes, with adults aged 60 and over accounting for over 95% of deaths in Europe (World Health Organization Europe, 2020). Approximately half of all COVID-19 fatalities in Europe occurred in residential care settings. Despite the fact that early on in the pandemic older people were identified as among those most at risk, older persons have rarely been prioritised in subsequent policies (UN, 2020). As stated by Dr Hans Henri P. Kluge, the WHO Director for the European Region, ‘this pandemic has shone a spotlight on the overlooked and undervalued corners of our society’ (WHO Europe, 2020). Indeed, the coronavirus crisis has exposed the extent to which ageism, including age-based discrimination and stigmatisation of older adults, is entrenched in policies, institutions, communities and the media, and ultimately, in society’s collective response to crises such as COVID-19 (Ayalon et al., 2021).

As with older people in residential care settings, those ageing in place who have had to shield at home have also faced particular challenges: health care denied for conditions unrelated to COVID-19; higher risks of violence, abuse and neglect; an increase in unemployment and poverty; the adverse impact on wellbeing, mental health and social connectedness and the trauma of stigma and discrimination (UN, 2020). But the lived experiences of older people during the pandemic have varied greatly, with research highlighting inequalities in the experience of health issues and problems relating to ageism, financial and digital exclusion, social isolation (i.e. the lack of social connections) and pressures on mental health (Ayalon et al., 2021; Ipsos Mori, 2020). Horton (2020: 48) makes the point that COVID-19 is not socially neutral, describing how: ‘Coronavirus exploits and accentuates inequality.’ Inequalities in later life are the product of cumulative advantage or disadvantage over time (Dannefer, 2003), with socio-economic precarity and systems of power contributing to experiences of discrimination, which have been further magnified in the context of COVID-19.

One important issue raised by research concerns the intensification of discrimination experienced by marginalised and stigmatised identities in the context of COVID-19. Initial findings from a web-based survey by Kneale and Bécares (2020), which explored the mental health and experiences of discrimination of LGBTQ+ people in the UK, found that almost one in five respondents had experienced some form of discrimination during the pandemic, with a ‘u-shaped trend in terms of age’, with the oldest and youngest LGBTQ+ groups at greatest risk of discrimination. This finding was supported by a survey by the LGBT Foundation (2020) in the UK, which highlighted the greater likelihood of isolation amongst older LGBT people (40% of survey respondents aged 50+ were living alone compared with 30% of all LGBT respondents). The survey noted: ‘LGBT older people who live in a world hostile to their identities may be reluctant to access support due to fears of encountering discrimination, further exacerbating this isolation and lack of support’ (2020: 9).

Research in the US has highlighted how persons who are both older and a member of a racial and ethnic minority group, particularly Black older people, have suffered disproportionately from COVID-19, when compared with younger persons and White people, as well as older White adults (Chatters et al., 2020). The authors use the concept of ‘double jeopardy’ to describe how racism and ageism together shape higher risks of COVID-19 exposure and disease, as well as poor health outcomes for older Black adults (Chatters et al., 2020). There is also strong evidence that increased ethnic inequalities in COVID-19-related complications and deaths exist in the UK (Bécares and Nazroo, 2020; Kirby, 2020; Platt and Warwick, 2021), with research reporting that people from ethnic minority groups (of all ages) are twice as likely to die from COVID-19, as well as having higher rates of hospitalisations and admissions to intensive care units (Razai et al., 2021). Further, findings from a Public Health England (PHE, 2020) report into disparities in the risks and outcomes of COVID-19 highlights that Black people in the UK had the highest diagnosis rate of any ethnic group, and a separate report found that South Asian people had the highest death rates according to hospital inpatient data in England (Harrison et al., 2020). Reasons for these disparities have been attributed to socio-economic inequalities and deprivation (PHE, 2020), and structural racism, with people from minority ethnic groups having poorer access to, and experience of, health care and treatment.

The pandemic has further highlighted systemic discrimination and vulnerabilities for older people with disabilities, those with long-term illnesses, and people with cognitive impairments such as dementia. A report by AGE Platform Europe (2020), focusing on older persons with disabilities in the European Union, highlighted a number of gaps in human rights protection exposed by COVID-19, including a lack of legislation that prohibits discrimination based on age and disability. The report discusses discrimination practices on the basis of disability and age in the triage process, and confinement; barriers in access to general health care and community-based support and social services; and reports of abuse (including fraud and financial abuse) of older people with disabilities (AGE Platform Europe, 2020). Research has further shown that older people living with dementia are not only at high risk of COVID-19 infection and more likely to experience severe virus-related outcomes, they are also at high risk of worsening psychiatric symptoms as a result of social isolation and confinement (Numbers and Brodaty, 2021).

Overall, older people with marginalised and stigmatised identities (race, ethnicity, gender, disability, LGBTQ+ and dementia) have experienced increased risks of domestic abuse, violence and reduced access to support (Ipsos Mori, 2020; Nazroo et al., 2020; UN, 2020). Adopting an intersectional lens helps illuminate how multiple factors combine or overlap to shape inequalities experienced by older people in the context of the COVID-19 pandemic (Scharf and Shaw, 2017). An intersectional approach draws attention to the diversity of the older population and the multiple forms of discrimination faced by older people such as women, ethnic minorities, LGBTQ+ people and those with long-term illnesses and disabilities. Addressing the unequal impacts of the pandemic, however, also requires an understanding of how underlying inequalities are generated across the life course and are amplified by institutional practices, discrimination and abuse. Furthermore, it requires consideration of how these intersect with spatial inequalities affecting the lives of older people. The next section examines the role of neighbourhood inequalities in the context of COVID-19, with a particular focus on the impact of the pandemic on urban neighbourhoods characterised by high levels of socio-economic deprivation.

## COVID-19 and neighbourhood inequalities

The coronavirus crisis has coincided with a period of deepening inequalities affecting many of the neighbourhoods in which older people live (Hambleton, 2020; Marmot et al., 2020). Marmot et al. (2020), for instance, traced changes in health inequalities over the period 2010–2020 in England, documenting the rise in deprivation affecting many parts of the country. The authors highlighted the problems facing what the researchers termed ‘left behind’ and ‘ignored communities’ experiencing the effects of long-term deprivation: ‘Over the last 10 years, these ... communities and areas have seen vital physical and community assets lost, resources and funding reduced, community and voluntary sector services decimated and public services cut, all of which have damaged health and widened inequalities. These lost assets and services compound the multiple economic and social deprivations, including high rates of persistent poverty and low income, high levels of debt, poor health and poor housing that are already faced by many residents’ (Marmot et al., 2020: 94).

Spatial disparities have been thrown into stark relief by COVID-19. Klugman and Moore (2020: 4) argue that ‘(t)he pandemic has exposed deep disparities in power and resources in cities, and revealed how existing forms of inequality can deepen the spread of global health and other crises’. The authors demonstrate how concentrations of poverty in certain neighbourhoods perpetuate disadvantage among the population. Such processes also explain the disproportionate impact that the pandemic is having on deprived urban areas already affected by cuts to public services, the loss of social infrastructure and pressures on the voluntary sector (Marmot et al., 2020; Yarker, 2019). Research in the UK found that people (of all ages) living in the most deprived areas were dying at *twice the rate* in the first wave of COVID-19, compared with those living in more affluent areas (Office for National Statistics [ONS], 2020). Similarly, a study of 10 major US cities (including New York, Boston, New Orleans and Los Angeles) highlighted a disproportionate burden of both infections and deaths in areas with a larger percentage of the population belonging to minority racial and ethnic groups and in neighbourhoods with higher rates of poverty (Adhikari et al., 2020).

In Britain, Beatty and Fothergill (2021) examined the impact of COVID-19 on the older industrial regions and former coal-mining areas, finding that the ‘cumulative death rates in older industrial towns and former coalfields was on average 20 per cent above the UK average’. They concluded that: ‘… the public health crisis in older industrial Britain was on average worse than in the rest of the country. Whether the scale of the crisis is measured in terms of the cumulative number of confirmed infections or deaths … the cities, towns and smaller communities of older industrial Britain dominated the list of worst-hit places’ (Beatty and Fothergill, 2021: 51).

In sum, the pandemic has exposed spatial disparities which have resulted in the poorest and most marginalised residents in cities being disproportionately affected by COVID-19. The next section of this paper explores how interrelated social inequalities at both the individual and spatial level are affecting the lives of older people living in low-income urban neighbourhoods during environmental and public health emergencies, with a particular focus on COVID-19.

## Locked down by inequality: Older people in times of crisis

The COVID-19 pandemic, and the wider governmental and societal response, have brought social and health inequalities into sharp focus. Older people have been disproportionately affected by COVID-19, but this reflects a more general pattern associated with environmental crises and disasters affecting urban areas (Phillips and Elliott, 2018). Evidence is already available for the disproportionate impact of hurricanes (Katrina in New Orleans in 2005 where 75% of those who died were 60 and over), and heatwaves (in Chicago in 1995, France, 2003). For example, Klinenberg (2002) examined the 1995 heatwave in Chicago, which during a 4-week period killed around 600 people, with 75% being aged 65 or over. As well as the immediate factors causing high mortality among older people, Klinenberg (2002: 55) pointed to structural features in the urban environment that discouraged older residents from seeking help, including barriers to physical mobility; neglect of local infrastructure; and the decrease in trusting, reciprocal relationships in areas with high levels of crime.

Ogg (2005) identified similar issues in his analysis of the 2003 heatwave in France that resulted in an estimated 15,000 deaths, most of whom were older people. The author cited several French studies that demonstrated that the highest mortality rates were in deprived urban neighbourhoods, particularly the Paris and Lyon conurbations. Ogg concluded that, as with the Chicago experience, the French heatwave raised important questions about the quality of life of older people living in densely populated urban areas: ‘these environments are often not adapted to the needs of older people and they can be one of the primary causes of social exclusion. Spatial and mobility-related aspects of citizenship are increasingly recognised as important dimensions of social inclusion … and older people in inner cities often face many disadvantages related to access to services’ (Ogg, 2005: 35).

The evidence reviewed suggests that older people living in socio-economically deprived urban areas are particularly disadvantaged in times of crises. But the COVID-19 pandemic has added extra pressures. In particular, older people who were required to shield or follow social distancing guidelines have experienced ‘a double lockdown’– suffering the effects of enforced social isolation (as a result of COVID-19-related instructions to self-isolate) whilst living in places affected by the loss of services and social infrastructure.

Research based on the English Longitudinal Study of Ageing (ELSA), carried out just before the pandemic hit, demonstrated a causal relationship between area deprivation and social exclusion in later life. The study revealed that older people living in deprived urban neighbourhoods had the *highest levels of social exclusion* compared with less deprived neighbourhoods, with evidence pointing to barriers experienced across a range of domains of exclusion such as access to services and amenities, social relationships and civic, cultural and leisure participation (Prattley et al., 2020). As a result, many older people living in deprived neighbourhoods are at risk of being isolated from the social networks of support and social connections that are essential to maintain a sense of wellbeing and belonging (Lewis, 2018; Yarker, 2019, 2020). The negative effects of social distancing on older people’s physical and mental health (Bailey et al., 2021) are thus likely to be further exacerbated by a lack of access to sources of social support linked to structural disadvantage, neighbourhood deprivation, cuts to local services and the voluntary sector as well as loss of vital social infrastructure. Consequently, older people who have been shielding in their homes in deprived neighbourhoods have experienced a ‘double lockdown’ as a result of the interrelated social and spatial inequalities associated with COVID-19.

Social distancing measures have also meant that many older people have spent more time in unsafe and hazardous homes. Ten million people are living in ‘non-decent homes’ in England, two million of whom are aged 55 and over (Centre for Ageing Better, 2020a). Non-decent homes are defined as being in a state of disrepair and/or having insufficiently modern facilities. Those who are more likely to live in poor housing are often the same groups who are vulnerable to COVID-19. The Centre for Ageing Better (2020a) report *Homes, Health and COVID-19* highlights the extent to which the pandemic has amplified housing-related health inequalities in the UK: first, through the acceleration of the virus in areas of poor housing; second, through measures to control the virus which have deepened health inequalities for those restricted to their homes. Ethnic minority groups have been amongst the worst affected by the deterioration of the housing stock. Data from the English Household Survey found that only 2% of White British households experienced overcrowding, compared with 24% of Bangladeshi households, 18% of Pakistani households and 16% of Black African households (Gov.UK, 2020). Given this context, shielding and self-isolation may be especially difficult for older people from ethnic minority groups, with the accompanying danger of risk of exposure to infection from COVID-19.

## Discussion: Age-friendly recovery planning and the future of the city

This paper has highlighted the impact of COVID-19 on the lives of older people and the communities in which they live. The issues discussed raise urgent questions about how to develop strategies to support marginalised groups of older people, especially those living in deprived urban neighbourhoods and trapped in poor quality housing. Bringing together perspectives from urban studies with research on ageing, this paper has offered a novel analysis of the ways in which COVID-19 has exposed, and exacerbated, inequalities in ageing as well as the social and spatial injustices in our cities.

Yet the pandemic provides an opportunity to engage in a radical rethink about the future shape of cities and communities, especially given changing demographics and work patterns (Buffel et al., 2020). This paper argues that recovery strategies need to focus on building back *fairer* cities and communities (Marmot et al., 2020), and that this will require embedding ‘age-friendly’ principles, based on a citizenship and rights-based narrative of ageing, and centred around values of equity, community empowerment and spatial justice. At a time when cities are starting to develop their long-term recovery strategies for ‘more inclusive, green and smart cities’ (Organisation for Economic Co-operation and Development [OECD], 2020), the importance of adding an ‘age-friendly’ lens is at least twofold: first, people aged 60 and over are the fastest growing cohort of urban populations (HelpAge International, 2016); and second, older people are often ‘erased’, or rarely incorporated into mainstream thinking and planning around urban environments (Buffel and Phillipson, 2018, 2019).

Given these arguments, post-COVID-19 recovery strategies aimed at cities and communities should build on at least six interrelated ‘age-friendly’ principles (Buffel et al., 2018; WHO, 2018): first, supporting the most vulnerable and prioritising resources in deprived areas; second, challenging the narrative on ageing and combatting ageism; third, promoting age inclusivity in the post-pandemic city; fourth, investing in community-based services, social infrastructure and green spaces; fifth, developing locally based partnerships across organisational boundaries; and sixth, involving older people in designing smart, liveable and resilient cities of the future (see Table 1).

**Table 1.** Six principles for integrating ‘age-friendliness’ into post-COVID-19 recovery strategies for cities and communities.

1. Supporting the most vulnerable and prioritising resources in deprived areas
2. Challenging the narrative on ageing and combatting ageism
3. Promoting age inclusivity in the post-pandemic city
4. Investing in community-based services, social infrastructure and green spaces
5. Developing locally based partnerships across organisational boundaries
6. Involving older people in designing smart, liveable and resilient cities of the future

### Supporting the most vulnerable and prioritising resources in deprived areas

Over the past decade, the age-friendly movement has been influential in developing insights about how to adapt urban environments to meet the needs of a growing and increasingly diverse ageing population (Rémillard-Boilard et al., 2021). Members of GNAFCC have also been at the forefront of developing initiatives to support the quality of life of older people during the pandemic. For example, age-friendly responses to COVID-19 have included initiatives to support social inclusion and participation, such as telephone call programmes to check on people who live alone, digital support services, campaigns to support older people with practical information about how to keep well in winter, initiatives to increase access to fresh foods or delivering food parcels to those in need and informative videos about service provision for individuals in ethnic minority communities for whom English is not a first language (American Association of Retired Persons [AARP], 2020; Centre for Ageing Better, 2020b; WHO, 2020).

However, many of the organisations that have developed such initiatives were in a financially precarious position as a result of economic pressures on local authorities and voluntary bodies – a situation that is likely to become even more severe given the impact of COVID-19 (Buffel et al., 2020). In the UK, to take one example, the voluntary sector lost an estimated £4.3 billion income during the first quarter of 2020, a reduction likely to have a significant impact on the support available to vulnerable groups (Weymouth, 2020).

The virus has, in different ways, amplified the challenges of providing collective support to marginalised populations, given a combination of increasing inequality and austerity. This has increased pressures on public and third sector services attempting to support older people, especially those at the highest risk, living in the poorest communities. This indicates the need for a targeted public health response, prioritising interventions and resources in areas of multiple deprivation, with a particular focus on specific groups within the older population: especially those from racial, ethnic and sexual minorities; those living alone; those living in areas with high population churn (e.g. with significant amounts of rented accommodation); and in areas with large numbers of multi-generational households.

### Challenging the narrative on ageing and combatting ageism

COVID-19 has not only taken a devastating toll on the lives of many older persons, it has also exposed a range of discriminatory practices against older adults. The number of deaths (direct and indirect) in care homes from COVID-19, and the delay in recognising the extent of the disaster, illustrate the extent of the crisis in social attitudes towards ageing. Ageism refers to ‘the stereotypes (how we think), prejudice (how we feel) and discrimination (how we act) directed towards people on the basis of their age’ (WHO, 2021: xv). Such discrimination often intersects with other forms of stereotypes and prejudice, such as sexism, racism and ableism. As well as discriminatory practices in relation to access to health services, physical isolation measures and strategies for lifting lockdown measures, ageism has also proliferated in news and media coverage of the pandemic, with dominant narratives around ageing centred around stereotypes of vulnerability and passivity. Older people have generally been depicted as a homogenous, frail group, presenting a burden and risk to other people.

Contrary to this discourse, evidence suggests that older adults have in fact made vital contributions to the crisis response, as caregivers, volunteers operating helplines, remotely helping children with their homework or by returning to work in the case of retired health-care workers (WHO, 2021). The British Society of Gerontology (BSG, 2020: 2) reminds us of some of the vital, but often ignored social roles that older adults play in society:

Older people participate in paid work, run businesses, volunteer, are active in civil society and the cultural life of communities, and take care of family members including parents, spouses/partners, adult children (especially those living with disabilities), and grandchildren. There are currently more than 360,000 people over 70 in paid work, including one in seven men between 70 and 75 and one in sixteen women. Almost one million people over the age of 70 provide unpaid care, including one in seven women in their 70s. One in five people aged between 70 and 85, over 1.5 million people, volunteer in their communities. Older adults should not be excluded but should be seen as a vital and necessary part of economic and community life. (BSG, 2020: 2)

The concern, however, is that the continuation of social distancing in some form, or subsequent waves of COVID-19 infections, are likely to further reinforce ageism and intergenerational divisions within communities. The Global Report on Ageism (WHO, 2021: 25) states that ‘the ageist narrative around younger and older people runs the risk of pitting generations against each other, as illustrated by the rapid spread of the hashtag “boomer remover” in reference to the virus severely affecting older adults’. Research evaluating twitter communication concerning older adults and COVID-19 found that nearly a quarter of all tweets had ageist or offensive content towards older people (Jimenez-Sotomayor et al., 2020). Given the risk of greater age segregation occurring as a result of COVID-19, it is essential to foster opportunities for greater contact between generations, challenge ageist stereotypes and highlight the diversity of experiences in later life (Buffel et al., 2014). The task for ‘age-friendly’ recovery strategies will be to support and strengthen ties across generations in order to develop an effective response to the present and future crises.

### Promoting age inclusivity in the post-pandemic city

COVID-19 raises significant question marks about the future characteristics of urban environments. Cities are viewed as benefiting from face-to-face contact and the resulting innovation and creativity (Wilson, 2020). Yet questions remain about the long-term impact of the pandemic. Urban life will continue, but the facilities, resources and spaces within it are likely to be experienced differently compared with the pre-pandemic era (Honey-Rosé et al., 2020).

One possibility for the future is that existing age-divisions will be reinforced as cities move out of economic and social lockdown. Glaeser (2021) suggests that the conditions of post-pandemic life may mean that cities become less expensive – as some groups move to the country and suburbs – but also riskier environments in which to live, reflecting economic pressures and recurring threats from pandemics. He concludes: ‘The biggest shift [for cities] will be the replacement of the old by the young who care most about socialising. It is the young who are least at risk of disease. It is young workers who have the most to gain by working and living in a city dense with opportunities to learn.’ Yet this seems a premature prediction to make: many European cities already have populations with between 20% and 25% aged 65 and over – a figure which looks set to increase rather than decrease (EPSON, 2019). Moreover, the qualities which attract older people to cities – such as good health care, accessible public transport and cultural facilities – will continue to be important in the years ahead.

There is no shortage of ideas to consider in respect of a future vision for the city. In 2013, the Royal Institute of British Architects (RIBA, 2013) outlined a range of plans for how ageing populations could engage in discussion about cities. Their ideas included: developing a new style of multi-generational homes – highly relevant given the problems of overcrowding highlighted by COVID-19; reviving the high street with a greater focus on public amenities rather than traditional shopping; expanding the University of the Third Age but with links to traditional universities; and promoting a network of green infrastructure across urban areas. These, and similar ideas, suggest that ageing populations – rather than being a negative force – could play a major role in rethinking the future of urban life.

### Investing in community-based organisations, services and social infrastructure

Research suggests that organisations embedded in local areas are particularly well placed to work with individuals and communities in order to identify those at risk of social isolation, and to engage them in finding solutions for developing new types of support (Durcan and Bell, 2015). Indeed, voluntary, community and social enterprise organisations have filled critical service provision gaps for marginalised populations during the pandemic, and will continue to play a vital role in addressing the needs of diverse vulnerable groups in the post-COVID-19 recovery (Hambleton, 2020). However, many of these organisations were already in financially precarious positions before the pandemic hit, with limited capacity to take on these roles (Buffel et al., 2020). This has been further compounded by cuts to social infrastructure in the form of community centres, leisure centres and libraries – the loss of which was already having a detrimental impact on those older people who are reliant upon their locality for social networks, sociality and support (Yarker, 2019, 2020). Research in the UK has shown that pre-pandemic cuts to local authorities have been especially high in more deprived neighbourhoods, leading to greater loss of services and social infrastructure in these areas (Marmot et al., 2020). This suggests that older people living in areas of intense deprivation have not only been disadvantaged in terms of accessing support during the lockdown, but that they are likely to be further disadvantaged and excluded from social support networks during any post-COVID-19 recovery.

Investing in community-based services and organisations which are providing vital social, psychological and practical support to marginalised and vulnerable groups will be a key task moving forward. Government allocations of funding to the voluntary and community sector will need to increase, and the resilience of neighbourhoods already weakened before the pandemic will require strengthening (Marmot et al., 2020). Alongside community-based capacity building and supporting local initiatives, investing in the physical and institutional infrastructure of cities which is crucial for the development and maintenance of social connections should also form a key part of recovery strategies to build back fairer communities. The social support generated in such third spaces has been found to be protective of health and wellbeing across the life course (Cotterell et al., 2018). Building on Klinenberg’s (2018) research on the importance of social infrastructure, Finlay et al. (2019) make the point that such community spaces ‘represent essential sites to address society’s pressing challenges, including isolation, crime, education, addiction, physical inactivity, malnutrition, and socio-political polarization’ (Finlay et al., 2019: 2). As cities and neighbourhood public spaces are gradually beginning to reopen following the pandemic, it will be important to ensure that older people, alongside other age groups, can safely access and regain use of the community spaces that are vital settings for their social life. Installing designated age-friendly benches in parks, ensuring seating to allow people to queue comfortably in shops and promoting accessible, green, safe and inviting public spaces, are just a few examples of how ‘age-friendly’ interventions may address the needs of different age groups.

### Developing locally based partnerships across organisational boundaries

Several pre-pandemic age-friendly initiatives have demonstrated the importance of building synergies and partnerships among multiple stakeholders and sectors – professional, academic, governmental and nongovernmental organisations – in developing new ways of researching and creating age-friendly environments *for, with* and *by* older people (Buffel, 2019; Rémillard-Boilard et al., 2021). The age-friendly cities and communities’ movement, in this respect, has a key role to play in breaking down silos between sectors and organisations by building on the assets and bringing together networks already present in cities, as well as creating new ones, in ways that benefit older people. Given the reality of economic austerity and competing demands for resources, strategic partnerships among public health professionals, local authorities, universities, housing providers, architects, community organisations and older people may be especially crucial to achieving success (Buffel and Phillipson, 2018; van Hoof et al., 2021). Mobilising a range of stakeholders from different sectors and disciplines and providing both top-down and bottom-up approaches in order to maximise the added value for each of the partners will be essential for realising the potential of the age-friendly movement in the post-pandemic city.

Hambleton (2020), in his book *Cities and Communities Beyond COVID-19*, develops the argument that the future development of cities will, to a large extent, depend on place-based collaborative leadership. The key challenge for post-COVID-19 strategy, the author argues, is ‘to recognise that we need to develop much more effective arrangements for anticipating and coping with complex threats – of whatever kind. […]. Enhancing place-based power and influence is critical, as it builds societal resilience’ (Hambleton, 2020: 166–167). Co-ordination of services at the local level and innovative collaborations within and across organisations are essential – at every level – to maximise collective efforts and make the most of the limited resources available. Local governments, despite financial pressures, have been on the front line in dealing with the COVID-19 crisis, working in partnership with local health authorities, community leaders and other local stakeholders. Local community-based efforts have been found to be particularly well placed to meet the needs of diverse vulnerable groups. Different types of support, such as advocacy, befriending and counselling, will need to be strengthened over the long-term. But given the extent of the crisis affecting communities, a broader range of activities at a neighbourhood level should be encouraged in the post-pandemic city, including: supporting the development of food co-operatives, low-cost home repair services, financial advice, re-invigorating ‘third spaces’ (such as cafés and community centres) and facilitating community leadership (Goff et al., 2020).

### Involving older people in designing smart, liveable and resilient cities of the future

Digital technology has played a crucial part in the various responses to the pandemic, driving health and safety interventions, helping people access health care, education and work and enabling people to connect with each other. Digital technology has also played an important role in supporting vulnerable groups in cities, for example through supporting online shopping and providing opportunities for community groups to meet online and organise support for those in need (Marston et al., 2020).

On the one hand, it will remain critically important to provide sufficient non-digital routes to communication, participation and access to services (e.g. via telephone and distribution of key messages in paper form) for those who lack digital skills or are facing digital exclusion – an estimated 5 million people over the age of 55 in the UK have no online access (Centre for Ageing Better, 2020c), with older women, those in poor health, and those in poorer financial circumstances the least likely to have internet access (Matthews et al., 2019). On the other hand, national and local authorities should commit to universal access to the internet, by working to expand access to broadband and data or telephone packages, particularly for individuals and families on low incomes who are most likely to be digitally excluded (Centre for Ageing Better, 2020c). But ensuring that digital services are accessible will not be sufficient if people experience barriers or cannot use technology. Therefore, government and service providers should also invest in schemes to support people in mid to later life to get online and to remain digitally included throughout their later life, including after the onset of poorer health or impaired physical ability, especially as some of these individuals might benefit the most from some of the services that digital technology can provide (Matthews et al., 2019).

Marston et al. (2020: 31) developed the ‘Concept of Age-Friendly Smart Ecologies’ (CASE) offering a useful framework for cities ‘to take an agile approach and work together in a locality approach to adopt and implement improvements […] by employing innovative technologies’. ‘Smart city’ initiatives such as digital portals and apps, electric vehicles and artificial intelligence technology have great potential to improve the ‘age-friendliness’ of communities through their focus on developing innovative technologies to improve and enhance liveability, independent living, quality of life, resilience and economic growth. However, as Marston and van Hoof (2019) have pointed out, the ‘smart’ city and ‘age-friendly’ projects have remained largely disconnected, with the risk of both movements being weakened by operating separately from each other. Encouraging links between different urban projects and movements may encourage opportunities to expand the range of age-friendly interventions in the post-pandemic city. For example, ideas from the smart and sustainable cities movement around increasing energy efficiency, supporting alternatives to cars and reducing pollution, should also be a central part of making cities inclusive for all age groups. Engagement with this type of work has the potential to produce further resources for the age-friendly movement and add to the sustainability of existing projects (Phillipson and Buffel, 2020). It may also enhance a co-production approach, bringing together businesses, urban planners, policy-makers, technologists and older residents in designing and re-imagining the smart, liveable, sustainable and age-friendly city of the future. A key element in this will be to ensure that older people are centrally involved in developing emergency responses and preparedness plans:

Disaster risk reduction and preparedness plans need to be ‘older persons friendly and inclusive’ to prevent and mitigate the potentially devastating implications of emergency crises among them. The challenge is not only to protect older persons and ensure essential services provide for their needs, as part of the emergency response and recovery after crises, it is also to account for the diversity of this population group, recognize their capacities and harness their experience to maximize the preparedness for and minimize the impact of emergencies. (UNECE, 2020: 1)

## Conclusion

COVID-19 is a defining public health challenge for the 21st century. However, this is being amplified in the context of widening inequalities in countries such as the UK, for a variety of reasons: first, the social fabric has been drastically weakened through a decade of reductions in public expenditure – most notably in public health-related areas. Second, through pressures arising from an ageing population, especially with increases in the numbers of those 80 and over – a group disproportionately affected by serious illness and death related to COVID-19. Third, the impact of increased inequality in society – to repeat a statistic cited earlier: people (of all ages) living in the poorest parts of England and Wales were dying at twice the rate from the disease compared with those in more affluent areas (ONS, 2020); and ethnic minority communities have been amongst the most severely affected by the pandemic (Bécares and Nazroo, 2020).

The issues identified underline the need for implementing an age-friendly recovery plan, such as the one outlined in this paper, with the values of community empowerment, anti-discrimination and anti-racism at its heart. COVID-19 must cause a rethink in the kind of urban infrastructure needed to support vulnerable populations in times of crisis. For older people – as with other groups – the consequences of the pandemic have been to weaken the ‘informal’ networks which sustain everyday life. But the effects have been amplified for those living in socio-economically deprived neighbourhoods where austerity and now COVID-19 have had the greatest impact. The task now is to find urban solutions to the issues posed by the pandemic, and to ensure that older people are active participants in developing the new public health policies necessary for the years ahead.
